# XMU-MP-1 induces growth arrest in a model human mini-organ and antagonises cell cycle-dependent paclitaxel cytotoxicity

**DOI:** 10.1186/s13008-020-00067-0

**Published:** 2020-09-17

**Authors:** Ellen Mitchell, Charlotte E. L. Mellor, Talveen S. Purba

**Affiliations:** Centre for Dermatology Research, University of Manchester & NIHR Biomedical Research Centre, Manchester, M13 9PT UK

**Keywords:** XMU-MP-1, Hippo, YAP1, Cell cycle, Proliferation, Hair follicle, MST1/2, Aurora B, Chemotherapy, Alopecia

## Abstract

**Background:**

XMU-MP-1 is an inhibitor of the Hippo pathway kinases MST1/2 and has been shown to promote the downstream activation of the pro-proliferative, pro-regenerative and anti-apoptotic transcriptional regulator YAP1. We tested whether XMU-MP-1 can activate YAP1 in a model human mini-organ, namely the hair follicle, to determine whether it can be pharmacologically exploited to promote regeneration in the hair follicle as a novel strategy to treat pathological hair loss disorders.

**Results:**

XMU-MP-1 treatment inhibited MOB1 phosphorylation but did not increase active YAP1 in the hair follicle. Rather than promote proliferation, XMU-MP-1 serendipitously decreased the number of Ki-67+, EdU+ and phospho histone H3+ hair matrix keratinocytes and antagonised the cytotoxic effects of paclitaxel.

**Conclusions:**

XMU-MP-1 perturbs epithelial cell cycle progression in a model human mini-organ. This may arise as an off-target effect, especially when XMU-MP-1 has been described to strongly inhibit 21 additional kinases beyond MST1/2. Therefore, whilst these effects may be dependent on tissue context, researchers should exercise caution when interpreting the effects of XMU-MP-1, especially in tissues with actively proliferating cell populations.

## Background

Hair loss can be highly distressing for affected individuals, and efficacious prevention and treatment options are limited. Therefore, there is an urgent need to identify new therapeutic approaches that either protect the hair follicle from damage or promote regeneration in the context of potentially permanent hair loss disorders associated with cancer treatments and inflammatory conditions [1–3].

The Hippo pathway, as a key regulator of growth and differentiation, has emerged as an attractive target in translational medicine to promote tissue repair and regeneration [4–6]. The Hippo pathway is a kinase cascade, whereby the MST1/2 kinases activate the LATS1/2 kinases, which in turn phosphorylate and inactivate the transcriptional regulator YAP1 [7]. YAP1 is pro-regenerative and anti-apoptotic, and its inactivation via Hippo signalling prevents the transcription of genes associated with proliferation and survival [7].

Previous work has identified the small molecule XMU-MP-1 as a potent inhibitor of MST1/2. Accordingly, it has been reported that XMU-MP-1 increases YAP1 activation and decreases apoptosis, which promotes repair and protection against damage in models of liver injury and experimentally-induced colitis [8]. However, supporting KINOMEscan profiling data within this same study reported that XMU-MP-1 has a strong affinity to inhibit an additional 21 kinases [8]. Despite this, follow-up studies have since claimed to have successfully utilised XMU-MP-1 to specifically target Hippo signalling, highlighting protective applications against early brain injury [9] and cardiac pressure overload [10].

As such, we sought to determine whether XMU-MP-1, through its putative growth promoting properties, could be exploited to protect against damage and promote regeneration in the hair follicle as a novel means to therapeutically manage pathological hair loss disorders. Using an ex vivo human hair follicle mini-organ culture model [11], we treated terminal human anagen VI hair follicles with XMU-MP-1. Anagen is the active growth phase of the hair cycle, whereby hair follicles show extensive keratinocyte proliferation and differentiation to support hair shaft production which can last for years [12]. To dissect the potential growth promoting effects of pharmacological Hippo pathway inactivation, we analysed cell cycle parameters in proliferating human hair follicle matrix keratinocytes using in situ methods previously established and routinely utilised in human hair research [13].

## Results

### XMU-MP-1 perturbs cell cycle progression in proliferating human hair matrix keratinocytes

**Fig. 1:**
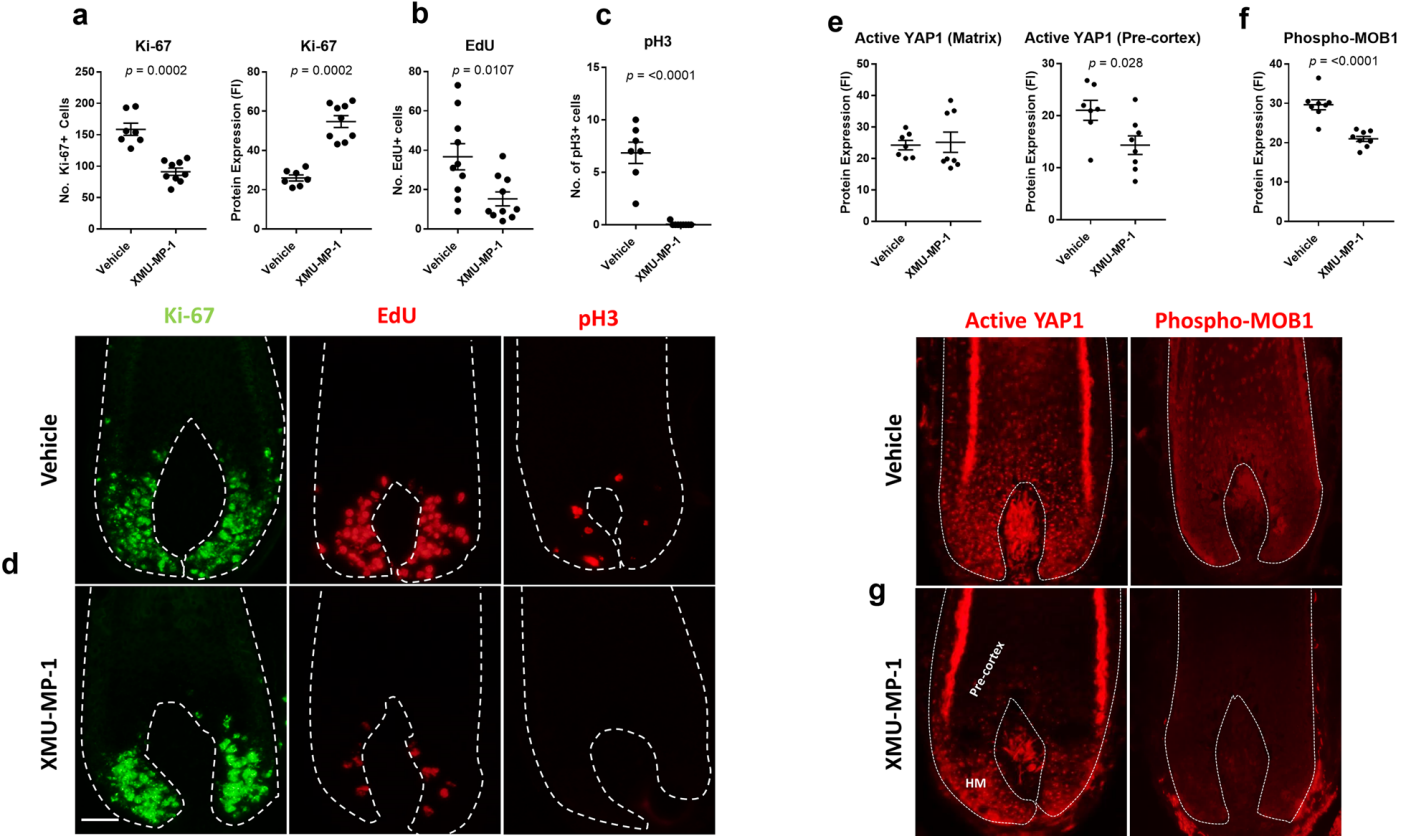
XMU-MP-1 perturbs cell cycle progression in proliferating human hair matrix keratinocytes. **a** Treatment of human hair follicles with 3 µM XMU-MP-1 for 24 h significantly decreases the number of Ki-67 + cells in the hair matrix, whilst simultaneously increasing Ki-67 protein expression (*p *= 0.0002 for both analyses). Mann–Whitney test performed on 7–9 hair follicles from 3 donors. **b** XMU-MP-1 significantly (*p *=0.0107) decreases the number EdU + cells (S-phase). Unpaired t-test performed on 10 hair follicles per group from 3 donors. **c** XMU-MP-1 significantly (*p *≤ 0.0001) decreases the number of mitotic phospho histone H3 (pH3) + cells. Mann–Whitney test performed on 7–9 hair follicles from 3 donors. **d** Immunofluorescence images show that XMU-MP-1 perturbs markers of cell cycle progression in the human hair matrix. 50 µm scale. **e** XMU-MP-1 does not affect the immunoreactivity of active YAP1 in the proliferative hair matrix but significantly (*p *=0.028) decreases active YAP1 immunoreactivity in the hair follicle pre-cortex. Mann–Whitney test performed on 7-8 hair follicles from 3 donors. **f** XMU-MP-1 significantly (*p *≤ 0.0001) decreases phospho-MOB1 (Thr35) immunoreactivity in the hair follicle matrix and pre-cortex. Unpaired t-test performed on 9–10 hair follicles from 3 donors. **g** Immunofluorescence images show that XMU-MP-1 selectively decreases active YAP1 immunoreactivity in the hair follicle pre-cortex and decreases phospho-MOB1 (Thr35) immunoreactivity in the hair follicle matrix and pre-cortex. Error bars represent standard error. *FI* Fluorescence intensity, *HM* Hair Matrix

To first determine if XMU-MP-1 augments proliferation in the human hair follicle, we treated hair follicles in ex vivo organ culture [11] with 3 µM XMU-MP-1 for 24 h and systematically dissected the cell cycle effects on anagen VI hair matrix keratinocytes [12, 13]. We found that the total number of Ki-67+ hair matrix keratinocytes was decreased in treated follicles (Fig. 1a, d), suggesting that XMU-MP-1 perturbs the cell cycle. Conversely, this coincided with an increase in the intensity of Ki-67 immunoreactivity in the hair matrix (Fig. 1a, d), indicating increased Ki-67 protein expression within the reduced fraction of proliferating cells. The number of cells in S-phase (EdU+) (Fig. 1b, d) and the number of mitotic phospho histone H3 (pH3)+ cells in the hair matrix was also significantly decreased (Fig. 1c, d). Together these results show that XMU-MP-1 disrupts normal cell cycle progression in a highly proliferative model human tissue system.

### XMU-MP-1 does not significantly increase active YAP1 in the human hair follicle matrix

We next explored the effects of XMU-MP-1 treatment on Hippo pathway proteins downstream of MST1/2. Active YAP1 is abundantly expressed in the human hair follicle, indicating functional importance to hair growth (Fig. 1e, g). XMU-MP-1 treatment did not significantly affect the level of active YAP1 staining in proliferating hair matrix keratinocytes (Fig. 1e, g). However, active YAP1 staining was selectively decreased in cell cycle-arrested terminally differentiating keratinocytes of the pre-cortex and hair shaft (Fig. 1e, g) [12]. This indicates that XMU-MP-1 does not effectively enhance active YAP1 in the hair follicle and may even disrupt active YAP1 in differentiating keratinocytes.

Next, we looked at phospho-MOB1 (Thr35) staining. In the Hippo-YAP pathway, LATS1/2–MOB1 is phosphorylated and activated by MST1/2-SAV1, which in turn inactivates YAP1 [7]. XMU-MP-1 treatment significantly decreased phospho-MOB1 staining in the hair matrix (Fig. 1f, g), which is in accordance with the successful inhibition of MST1/2. However, this did not result in a concomitant increase in active YAP1 staining or proliferation.

### XMU-MP-1 antagonises cell cycle-dependent paclitaxel cytotoxicity

**Fig. 2:**
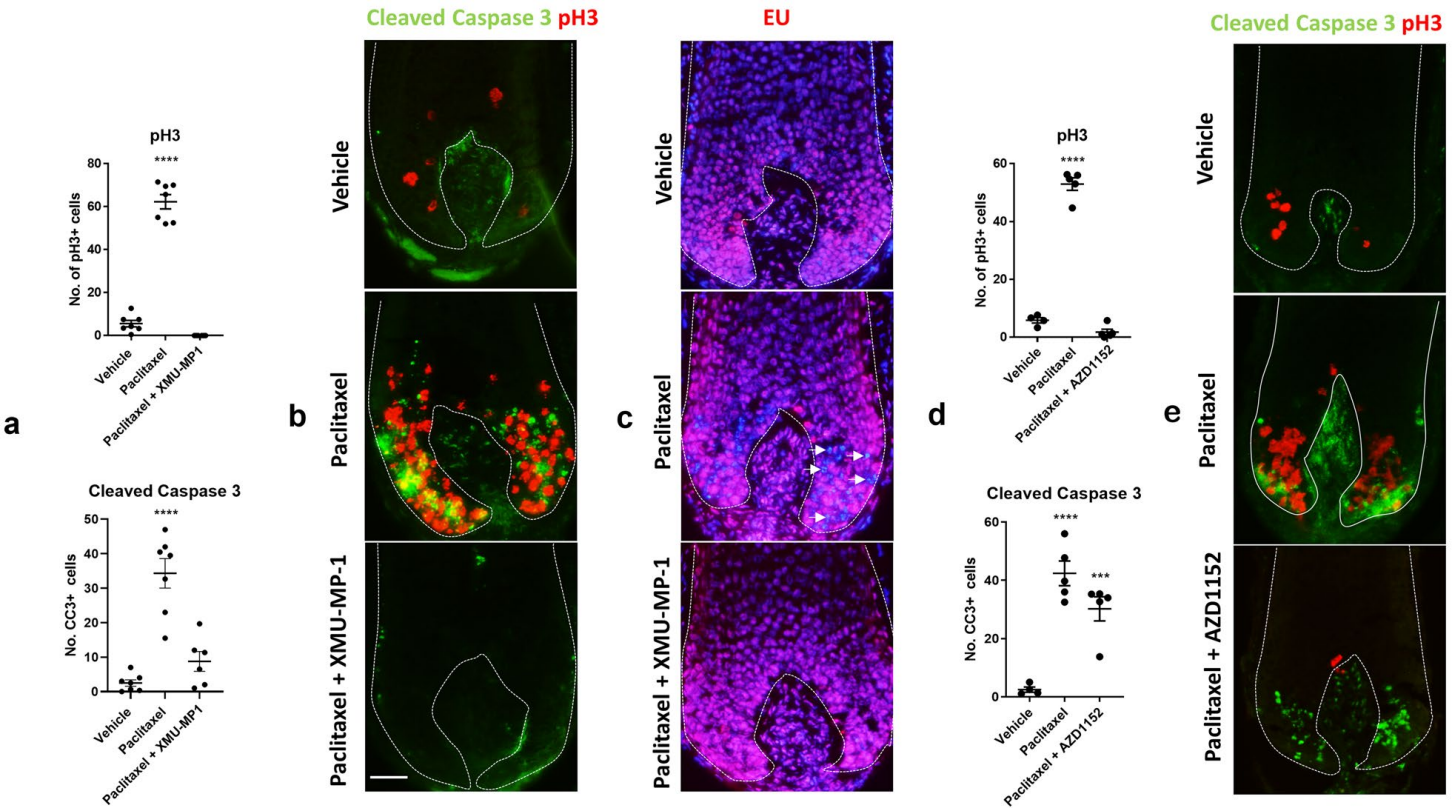
XMU-MP-1 antagonises cell cycle-dependent paclitaxel cytotoxicity. **a**, **b** XMU-MP-1 blocks the accumulation of phospho histone H3 (pH3)+ and cleaved caspase 3+ (CC3) cells in the hair matrix seen following paclitaxel treatment [2]. 50 µm scale. Analysis conducted using 4-7 hair follicles per condition from 2 donors. **c** Paclitaxel blocks nascent RNA synthesis, as visualised by 5-ethynyl uridine (EU) incorporation, in abnormally dividing hair matrix keratinocytes (white arrows) [2, 14]. This paclitaxel-induced block in RNA synthesis is prevented by XMU-MP-1 treatment. **d**, **e** Treatment with the Aurora B inhibitor AZD1152 also blocks the accumulation of pH3 + cells in the hair matrix following paclitaxel treatment, but results only in a trending reduction in the number of cleaved caspase 3 + cells, possibly due to the independent cytotoxicity of AZD1152 [16]. Analysis conducted using 4–5 hair follicles per condition from 1 donor. Adjusted *p* values = 0.0006 [***] and < 0.0001 [****]. Ordinary one-way ANOVA with multiple comparisons performed

To further probe the cell cycle-antagonising effects of XMU-MP-1, we co-cultured human hair follicles with XMU-MP-1 and the chemotherapy agent paclitaxel. We found that XMU-MP-1 blocked the cell cycle-dependent cytotoxic effects of paclitaxel, whereby the accumulation of pH3+ and cleaved caspase 3+ cells [2] was prevented (Fig. 2a, b). Moreover, XMU-MP-1 prevented a paclitaxel-induced nascent transcription block in abnormally dividing hair matrix keratinocytes (Fig. 2c) [2, 14]. This further supports that XMU-MP-1 can antagonise cell cycle progression in human hair matrix keratinocytes.

### Aurora B kinase inhibition blocks the paclitaxel-induced accumulation of phospho histone H3+ cells in the hair matrix

As it has previously been shown that XMU-MP-1 strongly inhibits 21 kinases beyond MST1/2, including TAO Kinases 1/2/3, JAK1, Aurora A/B and MEKK2/3 [8], we reasoned that the cell cycle arrest promoted by XMU-MP-1 in the hair follicle could be attributed to the off-target inhibition of kinases functionally important to cell growth.

The Aurora A/B kinases are critical mitotic regulators that phosphorylate histone H3, and the inhibition of these kinases can induce growth arrest [15–19]. When we independently tested the effects of Aurora B inhibition in the hair follicle for 24 h using 100 nM AZD1152 (Barasertib), we found that it blocked accumulation of pH3+ cells in the hair matrix following paclitaxel treatment (Fig. 2d, e). However, AZD1152 co-treatment only resulted in a trending decrease in the number of cleaved caspase 3+ cells relative to paclitaxel-only treated hair follicles (Fig. 2d, e), possibly due to the independent apoptosis-promoting effects of AZD1152 [16]. Whilst no direct causative connection can be made between the observed effects of XMU-MP-1 and AZD1152 in the hair follicle, these data highlight a potential mechanism for XMU-MP-1 that may be capable of impairing normal cell cycle progression.

## Discussion

In this study we show that, despite decreasing MOB1 phosphorylation, XMU-MP-1 does not significantly increase active YAP1 immunoreactivity or promote proliferation in the human hair follicle. Instead, XMU-MP-1 inhibited proliferation, which was subsequently capable of antagonising cell cycle-dependent chemotherapy-induced damage.

Given that XMU-MP-1 has been described to activate YAP1 via the specific inhibition of MST1/2 [8], we were initially surprised to find that XMU-MP-1 reduced the number of cycling Ki-67+ cells in the hair follicle matrix. Follow-up analyses further revealed that XMU-MP-1 significantly decreased the number of cells progressing through S-phase (EdU+) and decreased the number of pH3+ cells, suggesting that progression to M-phase and/or the mitotic phosphorylation of histone H3 was impaired.

To explore this further, we tested how hair follicles treated with paclitaxel responded to co-treatment with XMU-MP-1. Paclitaxel, which causes chemotherapy induced-alopecia, induces extensive mitotic defects and apoptosis in the hair matrix [2]. We found that XMU-MP-1 blocked cell cycle-dependent paclitaxel cytotoxicity in the hair follicle, as achieved previously via targeted G1 arrest using the CDK4/6 inhibitor palbociclib [2]. This data further supports that XMU-MP-1 can promote cell cycle arrest. Interestingly, XMU-MP-1 was effective against paclitaxel in the human hair follicle without the need for pre-treatment, as is required when targeting CDK4/6 [2]. This indicates that XMU-MP-1 might rapidly disrupt cell cycle progression into M-phase, where cells in the hair follicle are most vulnerable to taxanes [2].

Fan et al. describe, via their supporting KINOMEscan data, that XMU-MP-1 strongly inhibits numerous kinases beyond MST1/2 [8]. We therefore speculated that this lack of selectivity could account for the observations described in our study. In particular, we noted that XMU-MP-1 exhibits high-affinity binding with the Aurora A/B kinases, which control mitosis [17] and phosphorylate histone H3 [15]. It is therefore plausible that the effects exerted by XMU-MP-1 on the human hair follicle could be attributed to Aurora A/B kinase inhibition, especially when pharmacological Aurora kinase inhibition arrests growth [18, 19]. Indeed, we found that the independent treatment of human hair follicles with the Aurora B inhibitor AZD1152 also antagonised the cell cycle-dependent effects of paclitaxel in the hair follicle.

It should be noted that this experimentally replicated phenotype does not provide a causative connection between the effects of XMU-MP-1 treatment and targeted Aurora B kinase inhibition, especially when XMU-MP-1 could target numerous additional kinases that may influence the cell cycle. Therefore, whilst we can conclude that XMU-MP-1 perturbs cell cycle progression in the human hair follicle, the exact mechanism(s) by which this is exerted remains inconclusive.

In addition, we were unable to determine where exactly hair matrix keratinocytes are arrested in the cell cycle by XMU-MP-1 or AZD1152. Probing the cell cycle through in situ analyses is the preferred approach in human hair research [13]. This permits the study of hair matrix keratinocytes with respect to their spatial tissue context, and avoids the caveats associated with methods that require tissue disruption and analysis of cells outside of their native habitat [13]. However, in the absence of adequate multiplexing, analysis of cell cycle parameters in situ limits our ability to accurately quantify the cell cycle-phase distribution of a given population of cells. Future work would therefore benefit from the development and optimisation of imaging mass cytometry-based approaches to multiplex cell cycle-related readout parameters [20], which would allow researchers studying the human hair follicle to better analyse the cell cycle phase distribution of hair matrix keratocytes without sacrificing localisation data.

## Conclusions

XMU-MP-1 treatment did not significantly increase active YAP1 in a model human mini-organ; instead, it promoted epithelial cell cycle arrest which was capable of antagonising the cytotoxic paclitaxel. This may be due to the off-target inhibition of other functionally important kinases beyond MST1/2. Researchers should therefore exercise caution when utilising XMU-MP-1, especially when studying proliferative tissue systems. Furthermore, despite these unanticipated research outcomes, this work serendipitously identifies XMU-MP-1 as a fast-acting candidate small molecule that could be clinically exploited to antagonise cell cycle-dependent chemotherapy-induced damage.

## Methods

### Human hair follicle organ culture

Human scalp tissue was obtained with informed consent and stored in accordance with Human Tissue Act regulations.

Scalp tissue was dissected to isolate full length anagen VI hair follicles [11]. Hair follicles were cultured in 24 well plates, at 37 °C in 5% CO_2_/95% air, in hair follicle medium (500 µl per well) comprised of: Williams E medium supplemented with 10 µg/ml insulin, 100 U/ml penicillin, 100 μg/ml streptomycin, 2 mM l-glutamine and 10 ng/ml hydrocortisone. Hair follicles were incubated for 24 h in the presence of either vehicle (DMSO max. < 0.05%), 3 μM XMU-MP-1 (#S8334, Selleckchem), 100 nM AZD1152-HQPA (#ab142049, Abcam) and 100 nM paclitaxel (#1097, Tocris) with treatments delivered in combination where indicated. Where required, hair follicles were treated with either 20 μM 5-Ethynyl-2′-deoxyuridine (EdU) or 0.5 mM 5‐Ethynyl Uridine (EU), for 4 h and 2 h respectively, prior to completion of hair follicle organ culture experiments. Upon completion of experiments, hair follicles were frozen in embedding medium using liquid nitrogen and cryosectioned at 10 µm. Slides were stored temporarily at − 20 °C.

### Immunofluorescence

Slides with comparable tissue sections of the human hair matrix were selected for staining and allowed to air dry at room temperature for 10 min. Slides were subsequently fixed in ice-cold acetone for 10 min and left to air dry for a further 10 min. Each section was circumscribed using a hydrophobic barrier PAP pen (#H4000 Vector Laboratories).

Incorporated EdU/EU was detected as per kit instructions (Click‐iT™ EdU Alexa Fluor™ 594 Imaging Kit, #C10339, Thermo Fisher Scientific; Click‐iT™ RNA Alexa Fluor™ 594 Imaging Kit, #C10330, Thermo Fisher Scientific).

Primary antibodies were diluted in PBS (see below) and were applied to sections; slides were then incubated at 4 °C overnight. Slides were then washed three times in PBS for 2 min and secondary fluorescent antibody was applied at 1:200 in PBS for 45 min at room temperature (anti-mouse/rabbit Alexa Fluor 488/594). Slides were then washed three times in PBS for 2 min and treated with Hoechst 33342 (1:1000 in PBS) (#H3570, Thermo Fisher Scientific). Slides were finally washed in PBS for 2 min and mounted using aqueous mounting medium (#S3025, Dako).

### Antibodies

Anti‐Ki‐67 [SP6] (Abcam, ab16667) (1:50). Phospho-Histone H3 S10 (pH3) (#9706S, Cell Signaling Technology) (1:100). Cleaved Caspase-3 (#9661, Cell Signaling Technology) (1:50). Phospho-MOB1 (Thr35) (D2F10) (#8699, Cell Signaling Technology) (1:100). Anti-YAP1 antibody [EPR19812] (active, non-phosphorylated form) (ab205270, Abcam) (1:50). Alexa Fluor 488 and 594 secondary fluorescent antibodies (#A11005, #A11008, #A11037, Thermo Fisher Scientific) (1:200).

### Microscopy and analysis

Microscopy and imaging was performed using a BZ‐8000 fluorescence microscope (Keyence). Images were handled using ImageJ (NIH) and quantitative data was handled using GraphPad Prism 8 (GraphPad Software).

Fluorescence intensity and positive cell number was measured within the hair matrix between treatment conditions. Statistical testing employed Unpaired *t* test or the Mann–Whitney U test. D’Agostino & Pearson omnibus test was used to assess normality. For multiple treatment groups, Ordinary one-way ANOVA with multiple comparisons was performed.

## Data Availability

Data generated or analysed during this study not already included in this published article are available from the corresponding author on reasonable request.
